# Dynamics of Physiological, Biochemical and Psychological Markers during Single Session of Virtual Reality-Based Respiratory Biofeedback Relaxation

**DOI:** 10.3390/bs12120482

**Published:** 2022-11-28

**Authors:** Eglė Mazgelytė, Julija Zagorskaja, Edita Dereškevičiūtė, Tomas Petrėnas, Andrius Kaminskas, Jurgita Songailienė, Algirdas Utkus, Gintaras Chomentauskas, Dovilė Karčiauskaitė

**Affiliations:** 1 Department of Physiology, Biochemistry, Microbiology and Laboratory Medicine, Institute of Biomedical Sciences, Faculty of Medicine, Vilnius University, M. K. Čiurlionio st. 21, LT-03101 Vilnius, Lithuania; 2 Human Study Center, Trakų st. 8, LT-01132 Vilnius, Lithuania; 3 Department of Human and Medical Genetics, Institute of Biomedical Sciences, Faculty of Medicine, Vilnius University, M. K. Čiurlionio st. 21, LT-03101 Vilnius, Lithuania

**Keywords:** respiratory biofeedback, respiratory sinus arrhythmia, stress reduction, virtual reality

## Abstract

Psychological stress exposure is associated with long-lasting health effects including memory problems, depression, aches and pains, eating disorders, and alcohol or drug use. Thus, there is a need to develop effective stress management strategies that are easy to learn and practice. Respiratory biofeedback is an evidence-based stress management technique presenting breathing-related information to help subjects learn specific breathing skills for relaxation. It is suggested that the use of biofeedback techniques in conjunction with virtual reality makes biofeedback training an even more effective tool for stress management. The current study aimed to investigate dynamics of distinct stress indicators before, after, as well as during one brief virtual reality-based respiratory biofeedback session. Thirty-nine healthy volunteers participated in the study. Individuals provided their saliva samples and evaluated their mood status, fatigue, and strain level before and after the session. The subjects’ heart and respiratory rate, heart rate variability, and galvanic skin response measures were recorded during the session. The results showed that after single 12 min relaxation session, there was a significant decrease in salivary cortisol concentration, heart and respiratory rate, as well as decrease in skin conductance values. Self-reported strain, fatigue level, and mood status also significantly improved. VR-based respiratory-biofeedback-assisted relaxation sessions might serve as an effective stress management strategy, as even single session had positive effects on subjects’ autonomic nervous system (ANS) and hypothalamic-pituitary–adrenal (HPA) axis activity, as well as self-reported fatigue, strain level, and mood status.

## 1. Introduction

The term “stress” as it is currently used was conceived in 1936 by Hans Selye, who defined it as “the non-specific response of the body to any demand for change” [1]. The key components of the “stress system” are the hypothalamic–pituitary–adrenal (HPA) axis and the sympathetic nervous system (SNS). Stressors cause the production of corticotrophin-releasing hormone (CRH) in the hypothalamus, inducing the secretion of adrenocorticotropic hormone (ACTH) in the posterior pituitary and the activation of the noradrenergic neurons in the locus coeruleus/norepinephrine (LC/NE) system in the brain. The LC/NE system plays the main role in the immediate “fight or flight” response by releasing epinephrine and norepinephrine, while ACTH elicits the secretion of cortisol in the adrenal cortex. Normally, CRH and ACTH levels vary in a predictable circadian cycle and are suppressed by high concentrations of blood cortisol via negative feedback loop [2]. The acute-stress response is immediate and intense. When acute stress is severe or lasts a longer period of time, it might have deleterious consequences on health [3].

Chronic stress can cause many different symptoms: cognitive, which include memory problems, difficulty in concentrating, constantly worrying, racing mind; emotional: depression, anxiety, mood swings, irritation, abandonment; physical: pains and aches, diarrhea or constipations, chest pain, tachycardia, sickness, frequent colds; and behavioral: eating disorders, changes in resting hours, withdrawing from others, neglecting responsibilities, smoking, and alcohol or drug use in order to relax [2,4].

Fortunately, there are many evidence-based stress-reduction techniques which are easy to learn and practice. They include biofeedback, autogenic training, progressive muscle relaxation, diaphragmatic breathing, emotional freedom technique, relaxation response, guided imagery, transcendental meditation, and mindfulness-based interventions. They all can diminish bodily and mental tension, causing a decrease in disease symptoms, prevention of disease, and improvement of the patient’s quality of life [5].

Biofeedback, which is the main subject of this research, is a process which helps an individual to learn how to change physiological activity with the intention to improve health. Heart rate, blood pressure, and muscle tension are examples of physiological functions that people can learn to control [6]. During biofeedback training, precise instruments measure and give information to the user about distinct physiological stress/relaxation indicators such as brainwave or breathing patterns, heart function, muscle activity, and skin temperature. The presentation of the aforementioned information, often in conjunction with changes in thinking, emotions, and behavior, supports desired physiological changes. Over time, these alterations can be maintained without the continuous use of an instrument [7].

Based on the content of biofeedback, it can be categorized into several types: neurofeedback, respiratory, heart rate variability, galvanic skin response, blood pressure or thermal feedback, and electromyography [6]. These techniques have been successfully used for the treatment of headache [8], hypertension and type II diabetes [9], asthma [10], anxiety disorders [11], depression [12], as well as for the reduction of pain and mental stress during an early postpartum period [13].

Respiratory biofeedback systems measure and present breathing-related information to help users learn specific breathing skills for relaxation and stress relief [14]. Abdominal or diaphragmatic breathing is considered to be the best technique for subjects beginning to practice breathing exercises. During abdominal breathing, a person inhales through the nose and expands the abdomen slowly by gently pushing out and down as the oxygen fills the lower lung cavity. When the abdomen is full, the individual exhales through the nose and pulls the abdomen back, pressing the lungs from the bottom. Overall, the main purpose of diaphragmatic breathing is to fill up the lungs completely. The major advantage of this breathing is the invigoration of the abdominal muscles, as their persistent movements massage the internal organs and boost blood circulation [15].

In the past two decades, respiratory sinus arrhythmia (RSA)—a biomarker of parasympathetic-nervous-system-mediated cardiac control—has proved to present reliable information of emotion regulation [16]. Heart rate varies simultaneously with respiration: the inter-beat-interval (IBI), the time difference between two beat pulses, is shorter during inhalation and longer during exhalation [17]. This physiological phenomenon is called “respiratory sinus arrhythmia” [14]. In some studies, RSA biofeedback is considered to be a certain type of HRV biofeedback (HRV-BF), as the IBI shows heart rate variability (HRV) and assists individuals in controlling their breathing [14]. Previous studies showed that RSA biofeedback interventions effectively reduced resting heart rate, skin conductance level, and systolic blood pressure [18,19], lowered anxiety levels, and improved resistance to stressful situations [20].

It has been suggested that the use of virtual reality (VR) can make respiratory-based biofeedback techniques an even more powerful tool for coping with stress. VR can help the user to immerse completely into the biofeedback process by improving the visual attractiveness of biofeedback stimuli. Furthermore, the virtual environment is controllable and can be created both to maintain relaxation and to focus attention by using engaging environments [21,22].

Previous RSA and respiratory biofeedback examinations showed its effectiveness in relaxation; however, most of them focused on changes in single or several stress indicators during biofeedback training [18,19,20,23,24]. The aim of the present study is to investigate alterations and dynamics of distinct stress indicators before, after, as well as during the biofeedback session in virtual reality. We hypothesized that even a single session of VR-based respiratory biofeedback would have positive effects on average heart and respiratory rate, heart rate variability, galvanic skin response values, salivary cortisol levels, and the psychological state of study participants. Since the duration of the relaxation session in the current study was only 12 min, we assumed that this strategy might be a particularly attractive alternative for subjects who are not able to regularly practice strictly scheduled and time-consuming stress management programs.

## 2. Materials and Methods

### 2.1. Study Participants

Thirty-nine healthy volunteers (age 37.3 ± 6.7 years), comprising twenty-eight (72%) women and eleven (28%) men, were enrolled in the study. Participants were recruited at Human Study Center via pre-registration form. Each enrolled individual was contacted by experimenters by phone call. Subjects were excluded for medical conditions including chronic heart disease, metabolic and endocrine disorders, as well as mental diseases. Participants provided written informed consent before entering the study. Table 1 reports the sociodemographic and lifestyle characteristics of the study sample.

### 2.2. Procedure

To evaluate the change in psychological and physiological stress indicators as well as biochemical stress markers from before and after the relaxation session, individuals were required to attend the Human Study Center. The approximate duration of the visit was 1 h. Prior the relaxation session, participants completed a self-reported questionnaire on sociodemographic and lifestyle characteristics, filled out Perceived Stress Scale (PSS) and State-Trait Anxiety Inventory (STAI), as well as mood a status, fatigue, and strain rating form. After the completion of questionnaires, the first saliva sample was taken with the Salivette^®^ device. Subjects were asked to sit comfortably on a chair, their left arm with attached electrodermal activity electrodes was placed still on their left thigh during all biofeedback training session time. Individuals were explicitly instructed verbally just before the session following the script provided in Appendix A [25].

After the instruction and determination of the most suitable breathing rate for each individual, participants were exposed to 12 min of a virtual-reality-based respiratory-biofeedback-assisted relaxation exercise. At the beginning of the session participants were asked to breathe for a minute at 8, 7, 6, and 5 breaths per minute following the suggested wave in VR and in this way, the most beneficial respiratory rate was determined for each study participant. During the entire relaxation exercise the subject’s heart rate, respiratory rate, heart rate variability, and galvanic skin response measures were recorded. After the conclusion of relaxation session, individuals were asked to evaluate their mood status, fatigue, and strain level. Also, the second saliva sample was collected.

### 2.3. Measures

#### 2.3.1. Perceived Stress and Anxiety

Perceived Stress Scale (PSS) is a 10-item self-report measure assessing subjectively appraised stress over the past month. Participants were asked to rate each item on a Likert-type response scale ranging from 1 = never to 5 = very often. Higher overall score indicates a greater perceived stress level.

State and Trait Anxiety subscales of the STAI were used as a subjective measure of anxiety. The S-Anxiety scale evaluates the current state of anxiety while the T-Anxiety scale assesses how subjects “usually” feel. Each STAI score ranges from 20 to 80, with higher scores indicating greater state and trait anxiety levels [26].

#### 2.3.2. Mood Status, Fatigue, and Strain

Participants were asked to rate their mood status, fatigue, and strain on a scale from 1 to 5 developed by Human Study Center before and after the relaxation session. A rating of 1 indicated depressed mood, high fatigue, and strain, while 5 indicated good mood, low fatigue, and low strain level.

#### 2.3.3. Salivary Cortisol and Cortisone Levels

The samples were collected using Salivette^®^ (Sarstedt, Rommelsdorft, Germany) devices before and immediately after the relaxation session (pre- and post-session samples). The subjects were asked to restrain from alcohol consumption for 48 h, intense exercise for 24 h, eating, drinking (except water), smoking, and brushing their teeth or using dental floss for 1 h prior to saliva collection procedure. Saliva samples were stored at −80 °C till the analysis. Cortisol and cortisone levels were determined using liquid-chromatography-tandem mass spectrometry (LC-MS/MS) technique. The description of the sample preparation procedure and LC-MS/MS conditions are presented in our previous publication [27].

#### 2.3.4. Heart Rate, Respiratory Rate, and Heart Rate Variability Measurement

Heart rate (HR) measurements were made by a high-frequency infrared light earlobe Grove–Ear-clip Heart Rate Sensor, worn by each participant on the left earlobe and further processed and presented for virtual reality (VR) application by a microcontroller. Each pulse duration was determined and recorded by identifying the intervals between the highest blood oxygen saturation points. Heart rate was calculated by the formula:

$$HR=\frac{60,000}{HR_{durr}}$$

where *HR_durr_* refers to beat-to-beat intervals.

Respiratory rate measurements were made using a stretch belt in the abdominal area which measured the tension during inhalation and exhalation during the breathing cycle.

Heart rate variability (HRV) was evaluated by measuring time-domain HRV measures, including root mean square of successive RR interval differences (RMSSD) and percentage of successive RR intervals that differed by more than 50 ms (pNN50). Since artifacts or ectopic beats may affect the HRV values, the non-natural beats were removed using the quotient filter. The removal of non-natural beats using the quotient filter follows a simple rule: if the variation of two consecutive RRi values exceeds 20%, the filter removes the ectopic beat [28]. HR and HRV measurements were performed according to the guidelines from the Society for Psychophysiological Research [29].

*RMSSD* was calculated by the formula:

$$RMSSD=\sqrt{\frac{\sum_{i=1}^{N-1}{\left(RR_{i}-RR_{i+1}\right)}^{2}}{N-1}}$$

where *RR_i_* is the time interval between adjacent *R* waves, *RR*_*i*+1_ is the next *RR* interval, and *N* is the number of *RR* intervals.

*pNN50* was calculated by the formula:

$$pNN50=\frac{NN50}{NN}$$

where *NN50* is the number of the *NN* intervals with at least a 50 ms difference, and *NN* is the total number of the *NN* intervals.

#### 2.3.5. Galvanic Skin Response

Galvanic skin response (GSR) was measured as a digitized resistance—the average of one-minute activity level. GSR was calculated by the formula:

$$\mathrm{GSR}=V_{in}\;\cdot \left(\frac{V_{ref}}{1024}\right)$$

where *V_ref_* refers to the initial voltage, and *V_in_* refers to the output voltage.

The electrical resistance of the subject’s skin was measured via electrodes attached to the index and middle fingers of the person’s left hand. 10-bit digital resolution and 5000 mV initial analog voltage (*V_ref_*) were chosen for digitization of measurements. The measured change in skin resistance changes the resistance ratio of the resistor network, which affects the voltage displayed by the sensor at the output (*V_in_*). This, in turn, is digitized, thus obtaining the mentioned 10-bit signal. Neutral resistor network matching (no human skin connected to the sensor system) was selected at the 3418 mV point (digital result—700). When the change in skin resistance causes a 4.9 mV change in the analog sensor output, the digital value changes to 1.

### 2.4. Statistical Analysis

Statistical analysis was performed with R version 4.0.3 and IBM SPSS Statistics 24.0. Quantitative variables are presented as median (interquartile range) (IQR) or mean ± standard deviation (SD). A paired-samples Wilcoxon test (for non-normally distributed variables) or paired-samples t-test (for normally distributed variables) was used to analyze the differences in physiological and biochemical stress biomarkers, as well as psychological stress measures, before and after the relaxation session. Cohen’s d or r effect size was calculated for the change in each psychological, biochemical, and physiological stress indicator. Cohen’s d = 0.2 is considered a “small” effect size, 0.5 represents a “medium” effect size, and 0.8 a “large” effect size. The interpretation values for r effect size are: <0.3 (small effect), 0.3–0.5 (moderate effect), and ≥0.5 (large effect). For the comparison of physiological stress indicators before and after relaxation session, the average of the first and the last 2 min of recorded HR, RR, HRV, and GSR measures was used in the analysis. The impact of variation in pre-session values of physiological and biochemical stress measures was minimized by calculating the percentage change according to the following formula:

$$\Delta \%=\frac{\left(post-session-pre-session\right)}{pre-session}\times 100.$$

A single-sample t-test was employed to examine whether percentage changes in biochemical and physiological stress biomarkers in response to respiratory biofeedback-assisted relaxation technique were statistically significant. Linear mixed-effects models (*lme4* package in R) were used to examine the dynamics of physiological stress measures during the 12 min relaxation session. All models included fixed effects of time (1–12 min of relaxation session) and random effects for individual.

## 3. Results

### 3.1. Perceived Stress and Anxiety Levels in the Study Sample

The analysis of PSS questionnaire showed that the majority of the study subjects considered their lives non-stressful (23.08%) or felt a moderate stress level (71.79%), and only two participants (5.13%) reported a high stress level during the previous month. The paired-samples t-test showed that mean ± SD scores of T-Anxiety were significantly higher compared with mean ± SD of S-Anxiety scores (42.18 ± 6.86 vs. 34.38 ± 12.54, *p* = 2.17⋅10^−5^). These results support the idea that under neutral (non-stressful) conditions, S-Anxiety scores tend to be lower or equal to T-Anxiety scores [26].

### 3.2. Determination of the Most Suitable Breathing Rate for Each Individual

**Figure 1:**
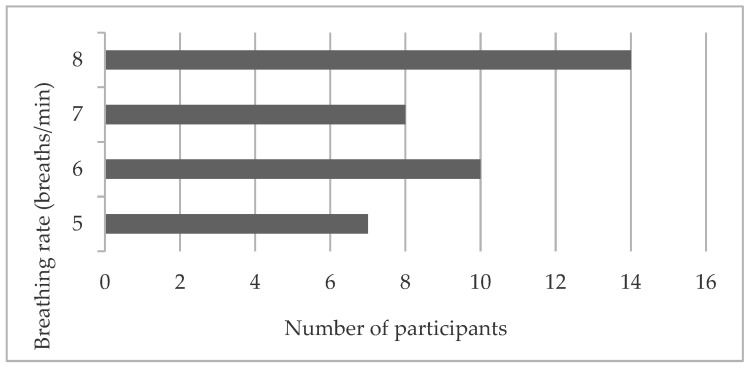
Distribution of the most suitable breathing rate in the study group.

Figure 1 shows the distribution of the most suitable breathing rate, which was determined at the beginning of the session, for each individual. The results indicate that for the majority of subjects (35.90%), the breathing rate of eight breaths per minute was the most beneficial.

### 3.3. Influence of Relaxation Session on Psychological, Physiological, and Biochemical Stress Indicators

The paired samples *t*-test showed significantly reduced strain and fatigue level as well as improved mood status after the relaxation session (Table 2). Similarly, reduced salivary cortisol secretion and a decreased cortisol-to-cortisone ratio was observed after the relaxation session. The decrease in total glucocorticoid (cortisol + cortisone) level reached a borderline level of statistical significance, while the change in cortisone concentration from before to after the relaxation session was not statistically significant (Table 3). Also, the relaxation session resulted in diminished heart rate, respiratory rate, and increased skin resistance values (Table 4). Analysis of percentage change values indicated a significant decrease in cortisol, cortisone, total glucocorticoid levels, cortisol-to-cortisone ratio, heart rate, and respiratory rate (i.e., negative percentage change), as well as an increase in skin resistance values (i.e., positive percentage change) (Table 5). No statistically significant change in time-domain heart rate variability measures (i.e., RMSSD, pNN50) were observed after the application of the relaxation technique (Table 4 and Table 5).

### 3.4. Dynamics of Physiological Stress Measures during Relaxation Session

Table 6 shows estimates for the linear mixed-effects model examining the dynamics of physiological stress measures during the relaxation session. Results indicate significantly decreasing heart rate and skin conductance values over time. However, no statistically significant change in breathing rate or heart rate variability measures were observed during the relaxation session.

## 4. Discussion

The study aimed to investigate the impact of a brief VR-based respiratory-biofeedback-assisted relaxation session on the ANS and HPA axis activity, as well as on subjective assessments of strain, fatigue, and mood. The results indicated significantly decreased strain and fatigue and improved mood after the single relaxation session (effect sizes ranged from small to moderate). Also, there was a significant decrease in cortisol levels from before to after the session, as well as in the cortisol-to-cortisone ratio (moderate and small effect size, respectively). Analysis of percentage change values of salivary steroid hormone concentrations revealed that relaxation session had a large effect size on the percentage change of cortisol level. The majority of physiological biomarkers (i.e., heart rate, respiratory rate, galvanic skin response) also enhanced relaxation state during the last 2 min compared with the first 2 min of the relaxation session. Linear mixed-effects models confirmed the aforementioned results, with the exception of non-significant change in breathing rate during the entire session.

Although it is agreed that slow breathing techniques promote autonomic changes by shifting ANS activity toward a parasympathetic predominance [30,31], results from previous studies examining the impact of these techniques on HR/HRV measures are conflicting [23,24]. Rockstroh et al. [24] investigated the effectiveness of a single-session VR-based HRV biofeedback exercise in a group of healthy young adults. The authors found a significant decrease in HR from mid- to post-relaxation session, without significant changes from pre- to mid-session. In contrast, RMSSD increased from pre- to mid- and decreased from mid- to post-session, with no differences from pre- to post-session values. It should be noted that the aforementioned changes in HR and RMSSD values during VR-based HRV biofeedback exercise were similar to alterations observed in traditional HRV biofeedback treatment with a graphical indicators group and control condition. A recent study conducted by Blum et al. [23] examined the feasibility of a VR-based diaphragmatic breathing biofeedback algorithm. Results showed that subjects assigned to the feedback group had lower mean respiratory rate and higher RMSSD values during a 7 min breathing exercise compared with the participants in the control group without biofeedback. Since both groups experienced the same virtual environment, the results supported the idea that biofeedback has an additional positive effect in learning to voluntarily control breathing rate and thus exert greater parasympathetic activity. Another study [32] investigated the effects of a single 60 min session of HRV-BF with paced breathing (6 breaths/min) training on time- and frequency-domain HRV indices and compared it with the results obtained from subjects who received autogenic training (AT). Analysis revealed higher SDNN, LF, lnLF, and LF/HF values, as well as lower respiratory rate after the HRV-BF training with large effect sizes (η_p_^2^ ranging from 0.32 to 0.36). Moreover, higher LF, lnLF, LF/HF, and a lower breathing rate post-training was observed in the HRV-BF group compared with AT group, while no differences in heart rate variability measures between groups were observed prior to the relaxation sessions. These findings indicate that slowing down the subjects’ breathing rate at approximately 6 breaths/min effectively increases parasympathetic tone and baroreflex gain. However, Van Diest et al. [33] showed that inhalation/exhalation ratio is a more important factor than respiration rate for inducing a self-reported relaxation state. Also, the study suggested that there is a combined effect of breathing rate and inhalation/exhalation ratio, as an increase in respiratory sinus arrhythmia was higher when participants were breathing at 6 breaths/min with a low inspiration/expiration ratio. Since the inhalation/exhalation ratio was not controlled in our study, this might explain the fact that no significant changes in RMSSD and pNN50 values were found in the current study. Thus, it would be interesting to examine the impact of respiratory-biofeedback-assisted relaxation training with controlled respiration rate and inspiration/expiration ratio (for example, an inhalation of 1.5 s and exhalation of 3.5 s) on heart rate variability measures.

Ambiguous results are presented in Tinga et al.’s [34] work. Their study examined the effectiveness of respiratory biofeedback in lowering subjective and objective arousal after stress. Respiratory biofeedback was compared to a control feedback placebo condition in which visual feedback unpaired to participants’ breathing was presented, and a control condition in which no feedback was provided at all. The decrease in heart rate and subjective tension was significantly stronger in the biofeedback group compared to the control feedback placebo; however, RMSSD and EEG theta-to-alpha ratio were higher in the control biofeedback group. Taken together, the results indicated that the control feedback placebo was superior to respiratory biofeedback in reducing arousal. The current findings highlight the importance of including a control feedback placebo condition when studying the effectiveness of biofeedback in order to establish the exact additional value of providing biofeedback [34].

The present study demonstrated that a VR-based respiratory-biofeedback-assisted relaxation approach reduced sympathetic activation as indicated by decreased skin conductance level at the end of the session. Similar findings were presented in the recent study [19], which aimed to evaluate the effectiveness of respiratory sinus arrhythmia (RSA) biofeedback training in a group of managers with high-level work responsibilities. The authors reported a significant decrease in skin conductance level after five weekly sessions of RSA-BF training and concluded that RSA-BF is an effective technique in reducing physiological arousal. To the best of our knowledge, only one study [35] evaluated the impact of rhythmic breathing on HPA axis activity by measuring morning salivary cortisol concentration before and after biofeedback-based stress management training which lasted for 28 days. Contrary to our study, Lemaire et al. [35] reported no statistically significant alterations in morning salivary cortisol level from pre- to post-training. It should be highlighted that the comparison between studies is not valid due to methodological differences, as we measured salivary glucocorticoid levels before and immediately after the single 12min relaxation session, while in the study conducted by Lemaire et al. [35], cortisol concentration was determined at baseline and after a 28-day relaxation trial.

The main strength of the study is that multiple stress-related biomarkers and indicators were used to evaluate the effectiveness of VR-based respiratory-biofeedback-assisted relaxation session. However, our study has several methodological limitations. It should be noted that there was no control group that did not participate in the VR-based relaxation session. Thus, the effectiveness of relaxation training should be confirmed in future studies with both experimental and control groups included. Since only young healthy volunteers, mainly women, were enrolled in the study, our work should be replicated in more heterogeneous populations. Moreover, data on menstrual cycle phase and sleep duration and quality was unavailable in the current study, and we were not able to include these potential confounding factors in the statistical analysis. Finally, a possible explanation of non-significant alterations in HRV measures during the session is the fairly short duration of the relaxation session, as well as the lack of information about HF component changes which reflect parasympathetic activity of the ANS. Therefore, the actual effect of the VR-based respiratory-biofeedback-assisted technique on subjects’ heart rate variability should be examined using both time- and frequency-domain HRV measures during longer duration training.

## 5. Conclusions

VR-based respiratory-biofeedback-assisted relaxation sessions might serve as an effective stress-management strategy, as even single session had positive effects on subjects’ autonomic nervous system and hypothalamic–pituitary–adrenal axis activity, as well as self-reported fatigue, strain level, and mood status. However, the effectiveness of the stress management technique developed in this pilot study should be confirmed in future studies utilizing randomized controlled trial design.

## Figures and Tables

**Table 1:** Sociodemographic and lifestyle characteristics of the study sample.

Variable	Mean ± SD or *N* (%)
**Gender**	
Women	28 (71.79)
Men	11 (28.21)
**Age (years)**	37.28 ± 6.98
**BMI (kg/m^2^)**	22.64 ± 2.97
**Smoking status**	
Non-smoker	33 (84.62)
Moderate smoker	5 (12.82)
Heavy smoker	1 (2.56)
**Exposure to environmental tobacco smoke**	
No	38 (97.44)
Yes	1 (2.56)
**Alcohol consumption**	
No	3 (7.69)
Yes (sometimes)	36 (92.31)
**Physical activity at work**	
Inactive	31 (79.49)
Active	8 (20.51)
**Leisure time physical activity**	
Inactive	6 (15.38)
Active	33 (84.62)

**Table 2:** Psychological stress indicators before and after relaxation session.

Variable	Pre-Session (Mean ± SD)	Post-Session (Mean ± SD)	*p*-Value	Effect Size (Cohen‘s d)
Strain	3.85 ± 1.06	4.38 ± 0.88	**0.001**	**0.556 (moderate)**
Fatigue	3.41 ± 1.14	4.13 ± 0.95	**<0.001**	**0.668 (moderate)**
Mood	3.64 ± 1.01	4.00 ± 1.08	**0.037**	**0.346 (small)**

**Table 3:** Biochemical stress markers before and after relaxation session.

Variable	Pre-Session (Median (IQR))	Post-Session (Median (IQR))	*p*-Value	Effect Size (Cohen’s d or r)
Cortisol (ng/mL)	2.24 (2.29)	1.85 (1.24)	**0.002**	**r = 0.469 (moderate)**
Cortisone (ng/mL)	11.79 (6.51)	11.68 (6.86)	0.166	r = 0.233 (small)
Cortisol + cortisone (ng/mL)	13.88 (9.62)	13.58 (7.97)	0.051	r = 0.291 (small)
Cortisol/cortisone	0.19 ±0.05	0.16 ±0.05	**0.008**	**d = 0.460 (small)**

**Table 4:** Physiological stress indicators before and after relaxation session.

Variable	Pre-Session (Mean ± SD or Median (IQR))	Post-Session (Mean ± SD or Median (IQR))	*p*-Value	Effect Size (Cohen’s d or r)
HR (bpm)	70.9 ± 6.75	69.35 ± 5.68	**0.002**	**d = 0.507 (moderate)**
RR (bpm)	7.5 (1.5)	7 (2)	**0.017**	**r = 0.430 (moderate)**
pNN50 (%)	23.63 ± 13.69	22.93 ± 13.95	0.604	d = 0.0838 (negligible)
RMSSD (ms)	42.97 ± 13.02	43.95 ± 15.46	0.984	d = 0.0321(negligible)
GSR	251.76 ± 123.99	320.38 ± 163.39	**<0.001**	**d = 0.795 (moderate)**

**Table 5:** Percent change in biochemical and physiological stress indicators.

Variable	Percent Change (Mean ± SD or Median (IQR))	*p*-Value	Effect Size (Cohen’s d)
Cortisol (%)	−24.00 ± 24.85	**<0.001**	**0.966 (large)**
Cortisone (%)	−10.11 ± 24.44	**0.018**	**0.413 (small)**
Cortisol + cortisone (%)	−11.70 ± 24.10	**0.006**	**0.486 (small)**
Cortisol/cortisone (%)	−12.06 ± 25.54	**0.008**	**0.472 (small)**
HR (%)	−2.23 ± 4.30	**0.002**	**0.519 (moderate)**
Respiratory rate (%)	−3.59 ± 6.36	**0.002**	**0.564 (moderate)**
pNN50 (%)	−2.52 ± 30.35	0.637	0.0830 (negligible)
RMSSD (%)	−0.25 ± 14.82	0.920	0.0166 (negligible)
GSR (%)	22.96 ± 27.31	**<0.001**	**0.841 (large)**

**Table 6:** Mixed-effects models of the dynamics of physiological stress measures during relaxation session.

Variable	Estimate	SE	*p*-Value
**Heart rate (bpm)**			
Intercept	70.99	1.03	
Time	−0.14	0.03	**<0.001**
**Respiratory rate (bpm)**			
Intercept	6.75	0.23	
Time	0.02	0.01	0.108
**Heart rate variability: RMSSD (ms)**			
Intercept	43.59	2.23	
Time	7.61×10^−4^	0.08	0.992
**Heart rate variability: pNN50 (%)**			
Intercept	23.99	2.04	
Time	−0.14	0.08	0.071
**GSR**			
Intercept	257.46	23.62	
Time	5.89	0.48	**<0.001**

## Data Availability

The data presented in this study are available on request from the corresponding author.

## Appendix A. The Instructions Provided to the Subjects Prior the Relaxation Session

*You will learn how to relax and manage stress with a help of breathing and biofeedback. For this purpose, we will firstly apply few sensors to your body: we will place a belt with a sensor around your waist, which will help us to monitor your breathing rate; we will put sensors on your two fingers on your left hand, which will help us monitor the conductivity of your skin; your heart rate will be monitored by a sensor attached to your left ear.*

*In addition to the physiological signals from your body that are monitored to assess how you are relaxing, two saliva samples will be taken before and after the relaxation session. Saliva samples will allow us to assess the change of the stress hormone cortisol. During the session you will be able to visualise how you breathe through virtual reality set. You will learn to manage your emotional state by changing your breathing and indirectly heart rate. These changes will be visible to you on VR in a way of changing waves.*

*Breathing is a powerful regulator of a human emotional state. Our heart rate increases and decreases along with breathing. When we inhale, our heart rate slightly increases, when we exhale, it decreases. The more harmoniously the heart rate changes along with the breathing, the more relaxed we are. However, in order to regulate your state with the help of breathing effectively, you need to breathe in a certain way—breath slowly, evenly, and exhale longer than inhale. In order for the session to be effective, we will firstly determine the most suitable breathing rate for you today. How do we make it? At the beginning of the session, we will ask you to breathe for a minute at 8, 7, 6 and 5 breaths per minute following the suggested wave in VR and in this way, we will find the most beneficial respiratory rate for you.*

*Once the relaxation part of the session starts, your breathing rate will be suggested in VR by the dot moving in the determined wave curve followed by a wide grey line. You will see notes “inhale” and “exhale” as well. You will also see another, thinner blue line—your actual breathing curve. Try to make your breathing line moving at the same pace as the wider grey line which shows recommended breathing rate for you. It does not matter where the blue line of your real breathing rate is in relation to the grey line in depth, it is important that both lines move in a synchronized way. If you breathe too deep or fast, you may become dizzy, and if this happens, breathe at a comfortable and normal breathing rate and depth for you. If you feel very uncomfortable you can terminate the session by raising your hand.*

*Try to breathe using your diaphragm. When you breathe by diaphragm, your belly rises during inhalation, and it lowers during exhalation. Please add your hand below your ribs and let’s try. When breathing try to exhale slower than you inhale. Your wave curve will change according to your breathing. The exemplary, broad grey line showing the suggested breathing rate, does not show the depth of breathing, it only shows the breathing rate. Try to breathe as deep as it is comfortable for you, and the grey exemplary breathing line will eventually adjust to your actual breathing depth. In addition to the two breathing lines, you will also see a third curve showing your heart rate. As you relax, the heart rate curve will begin to change following your breathing wave—it will rise with your breathing during inhalation and will drop with your breathing line during exhalation. Your goal is smooth breathing with longer exhalation than inhalation duration. While breathing you will also see notes at the top of your field of vision which will let you know how you are doing. Once your exhalation becomes longer than your inhalation, you will hear the sound “Om”. Both the notes and the sound you hear will help you understand how you are doing.*

*Now sit comfortably, place your hands on your thighs, put your feet flat on the floor. Keep your back straight, try not to lean on the back of the chair, and breathe calmly. You will also see instructions in VR goggles. If you experience any problems during the session, please raise your right hand at any time and I will come to you. Do you have any questions now?*
